# Fabrication of levofloxacin-loaded porcine acellular dermal matrix hydrogel and functional assessment in urinary tract infection

**DOI:** 10.1186/s12951-024-02322-w

**Published:** 2024-02-07

**Authors:** Yi Yang, Guang-Wei Yang, Jian-Juan Lu, Hao-Ran Chen, Ya-Qin Guo, Ning Yang, Yun-Zhu Zhu, Xiao-Qiang Liu, Ting-Ting Su, Yan-Yan Liu, Liang Yu, Ya-Sheng Li, Li-Fen Hu, Jia-Bin Li

**Affiliations:** 1 Department of Infectious Diseases and Anhui Center for Surveillance of Bacterial Resistance, The First Affiliated Hospital of Anhui Medical University, Jixi Road 218, Hefei, Anhui 230022 People’s Republic of China; 2 Anhui Province Key Laboratory of Infectious Diseases and, Institute of Bacterial Resistance, Anhui Medical University, Hefei, 230022 People’s Republic of China

**Keywords:** Bacterial cystitis, Porcine acellular dermal matrix hydrogel, Levofloxacin, Gut microbiota

## Abstract

Bacterial cystitis, a commonly occurring urinary tract infection (UTI), is renowned for its extensive prevalence and tendency to recur. Despite the extensive utilization of levofloxacin as a conventional therapeutic approach for bacterial cystitis, its effectiveness is impeded by adverse toxic effects, drug resistance concerns, and its influence on the gut microbiota. This study introduces Lev@PADM, a hydrogel with antibacterial properties that demonstrates efficacy in the treatment of bacterial cystitis. Lev@PADM is produced by combining levofloxacin with decellularized porcine acellular dermal matrix hydrogel and exhibits remarkable biocompatibility. Lev@PADM demonstrates excellent stability as a hydrogel at body temperature, enabling direct administration to the site of infection through intravesical injection. This localized delivery route circumvents the systemic circulation of levofloxacin, resulting in a swift and substantial elevation of the antimicrobial agent’s concentration specifically at the site of infection. The in vivo experimental findings provide evidence that Lev@PADM effectively prolongs the duration of levofloxacin’s action, impedes the retention and invasion of *E.coli* in the urinary tract, diminishes the infiltration of innate immune cells into infected tissues, and simultaneously preserves the composition of the intestinal microbiota. These results indicate that, in comparison to the exclusive administration of levofloxacin, Lev@PADM offers notable benefits in terms of preserving the integrity of the bladder epithelial barrier and suppressing the recurrence of urinary tract infections.

**Graphical Abstract:**

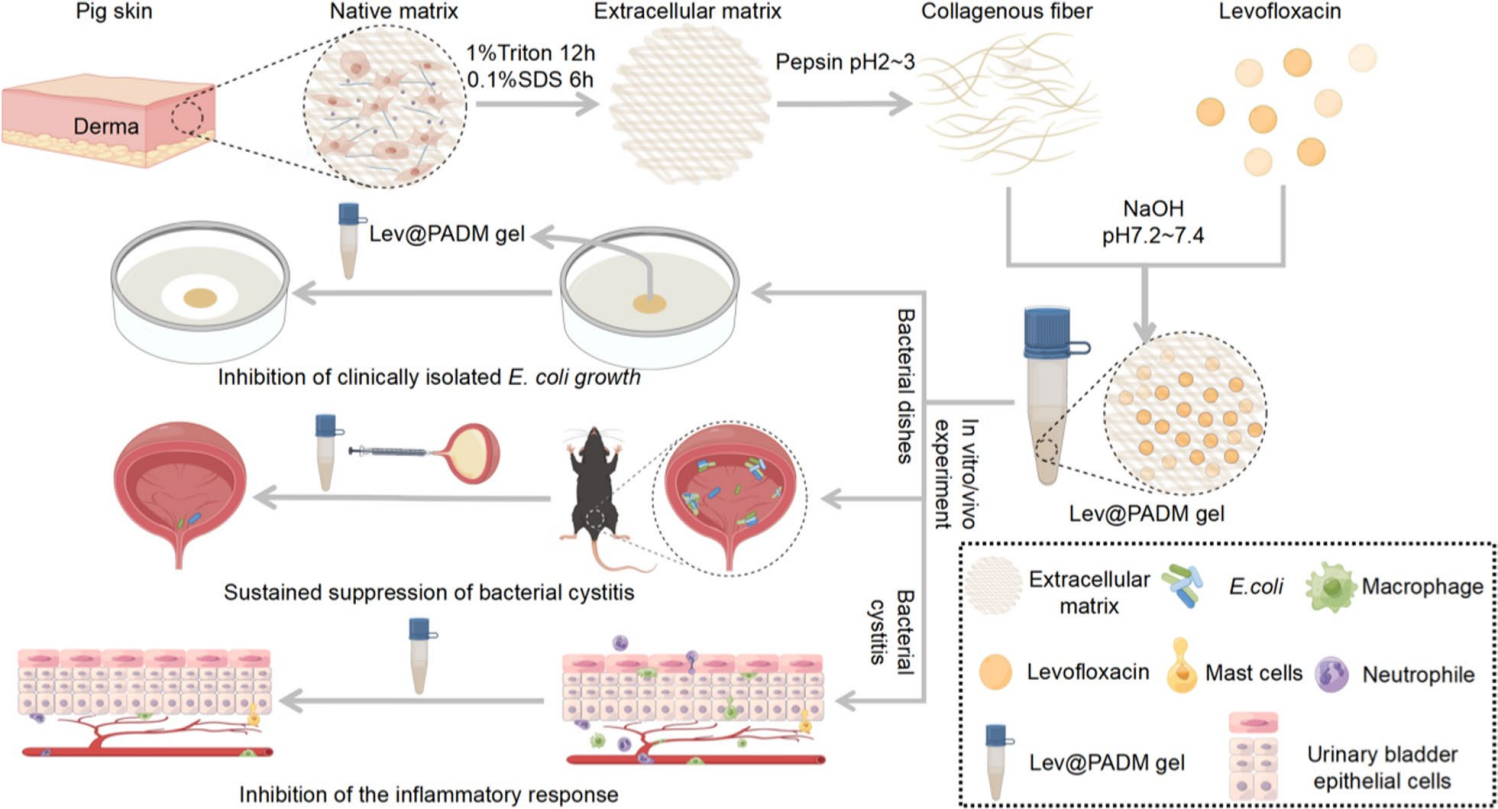

**Supplementary Information:**

The online version contains supplementary material available at 10.1186/s12951-024-02322-w.

## Introduction

Urinary tract infection (UTI) is a highly prevalent infectious disease in the global population, impacting an estimated 150 million individuals annually. This condition poses substantial economic and public health challenges [1, 2]. Among the various causative agents, *Escherichia coli*, *Pseudomonas aeruginosa*, *Staphylococcus aureus*, *Klebsiella pneumoniae*, and *Candida* species are the most frequently encountered pathogenic bacteria associated with UTIs. These bacteria possess the capability to persist within urothelial cells, and inadequate treatment can result in recurrent UTI episodes, thereby elevating the risk of secondary bloodstream infections [3].

The conventional treatment approach for urinary tract infections involves selecting beta-lactam agents, fluoroquinolones, trimethoprim-sulfamethoxazole, amoxicillin-clavulanic acid, or third-generation cephalosporins based on bacterial culture sensitivity results [4]. However, the treatment of urinary tract infections is faced with challenges due to the emergence of drug-resistant bacteria and the formation of intracellular bacterial reservoirs [5]. Additionally, the utilization of antimicrobial agents in this context is also limited by adverse drug reactions and drug interactions [6]. Moreover, a growing body of research indicates that antimicrobial drugs have the potential to disturb the ecological equilibrium of the gut microbiota and potentially enhance bacterial infections through modulation of the immune response [3, 7–9]. These findings emphasize the significance of formulating treatment strategies that are both safe and effective, utilizing current antimicrobial agents, to tackle these concerns and prevent and combat urinary tract infections.

The intravesical route of drug administration is a widely used method for treating urinary tract infections [10], interstitial cystitis [11], and bladder cancer [12] in a conventional manner. This targeted approach effectively reduces the systemic distribution of drugs, thereby minimizing the potential for systemic side effects, while simultaneously enhancing drug concentration and efficacy at the site of infection [13]. Nevertheless, the effectiveness of this delivery method is hindered by the intricate administration process and the limited duration of drug retention within the bladder [14].

Prior research has indicated that the utilization of hydrogel systems for intravesical drug delivery can effectively reduce drug dilution and enhance the mucoadhesive characteristics of drugs, resulting in prolonged drug retention and release within the bladder [13, 14]. However, conventional synthetic hydrogel materials, including polyethylene glycol-chitosan, alginates, thiolated succinic acid, and hyaluronic acid, demonstrate limited tissue compatibility due to their inherent toxic properties [15–19]. Additionally, crosslinking agents employed in hydrogel synthesis, such as acrylic acid, polyvinyl alcohol, and glutaraldehyde, also exhibit cytotoxic effects and are not readily neutralized or eliminated in vivo [18, 20–22]. In order to enhance the biocompatibility of hydrogels, scientists have devised extracellular matrix materials (ECMs) [23]. As previously documented, the complete ECM consists of proteoglycans, growth factors, and matrix cell proteins, such as collagen, elastin, laminin, fibronectin, hyaluronic acid, heparin, basic fibroblast growth factor (bFGF), vascular endothelial growth factor (VEGF), and platelet response proteins [24–26]. The creation of ECM hydrogels involves a self-assembly process primarily reliant on collagen, thereby demonstrating remarkable biocompatibility [27–29]. Presently, ECM hydrogels have found utility in vascular hemostasis [30], skin regeneration, and wound healing [31]. Nevertheless, there is currently a dearth of research concerning the application of ECM hydrogels in bacterial cystitis.

This study presents a novel approach in which levofloxacin, a widely employed broad-spectrum antibiotic for urinary tract infections, is non-covalently linked with dermal extracellular matrix hydrogel (porcine acellular dermal matrix (PADM)) for the purpose of treating *E.coli*-induced bacterial cystitis. By conducting comprehensive analyses of the physical property, cytotoxicity, and antibacterial efficacy of the antimicrobial hydrogel, we have established initial indications of the therapeutic potential of hydrogels in the management of bacterial cystitis. Moreover, our study has successfully demonstrated in a mouse model of bacterial cystitis that the antimicrobial hydrogel exhibits enhanced and sustained antibacterial effects, while also offering substantial protection for the gut microbiota when compared to the intervention involving levofloxacin alone. Additionally, the levofloxacin-PADM hydrogel formulation showcases notable innate immune modulation, surpassing the effects of levofloxacin alone. Consequently, the instillation of the levofloxacin-PADM composite hydrogel into the bladder presents a promising therapeutic approach for the management of urinary tract infections.

## Results

### Decellularization of porcine skin extracellular matrix

**Fig. 1:**
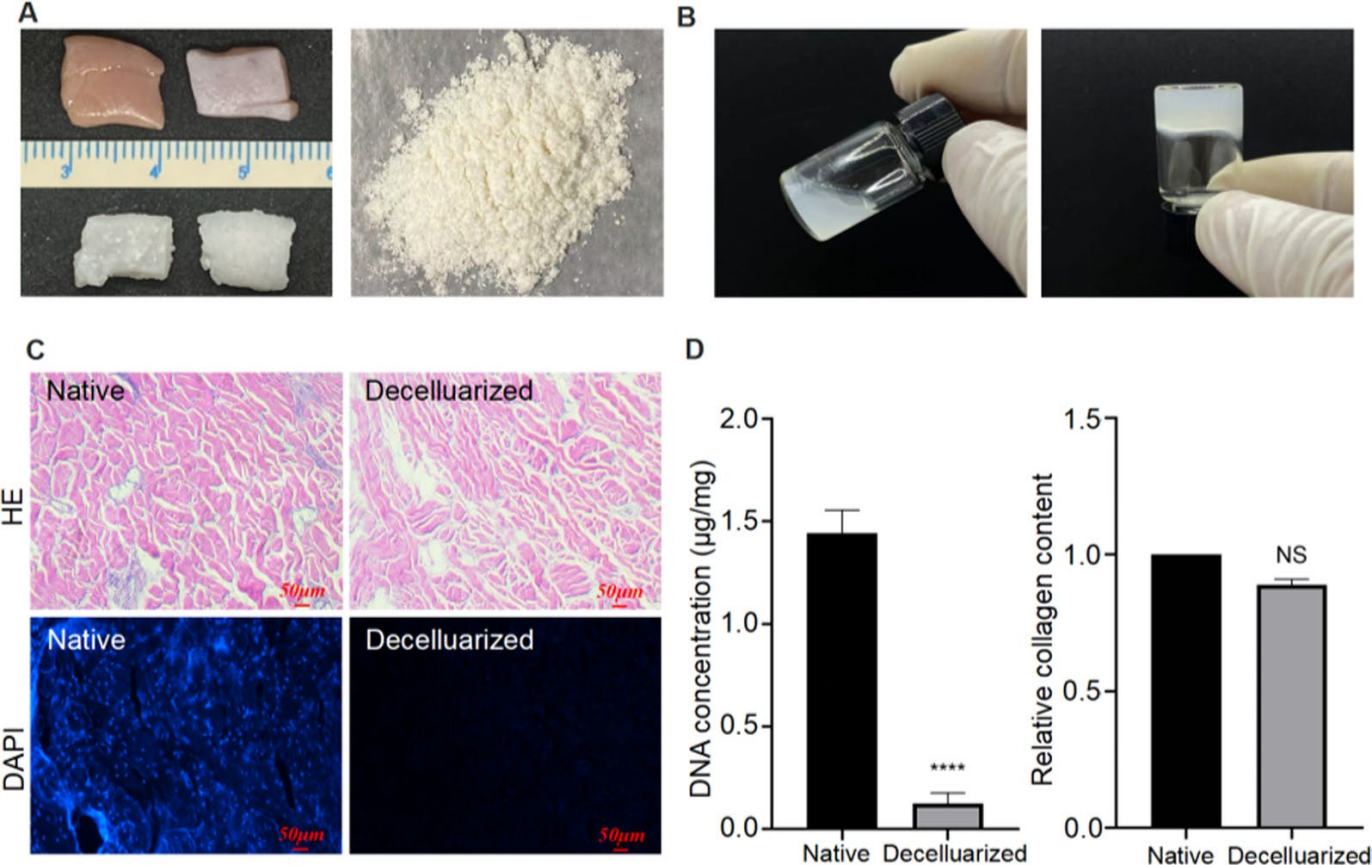
Decellularization of porcine skin and characterization of the treated material. **A** The top image shows natural pig skin, while the bottom image displays decellularized pig skin, which exhibits a milky white appearance. Additionally, the image on the right showcases freeze-dried and milled processed porcine exoskeletal matrix powder. **B** The left image depicts the porcine skin hydrogel precursor after undergoing pepsin digestion treatment for 24 h, while the right image displays the porcine skin hydrogel after pH adjustment. **C** Comparative pictures of porcine extracutaneous matrix before and after decellularisation under HE and DAPI staining; **D** Graph of changes in DNA content and collagen content of porcine extracutaneous matrix tissue before and after decellularisation. (ns: no significant; ****p < 0.0001)

PADM hydrogel is composed of a rich assortment of proteins, polysaccharides, growth factors, and matrix cell proteins, and it possesses the ability to form a gel without the requirement of cross-linking agents, thereby exhibiting exceptional biocompatibility [32–35]. The immunogenicity of RNA or DNA molecules as exogenous substances has always been a significant concern in drug delivery and gene therapy [36, 37]. Consequently, in this study, we initially subjected the porcine skin to decellularization treatment, resulting in a noticeable alteration in the visual appearance of the tissue from its original brown hue to white (Fig. 1A). Slices of the decellularized porcine skin tissue were subjected to staining with hematoxylin and eosin (HE) and DAPI. The results demonstrated the absence of cellular nuclei structures, while the PADM structures remained intact (Fig. 1C). Analysis of the DNA content in the decellularized porcine skin tissue exhibited a substantial reduction (p < 0.001), while the collagen protein content did not exhibit a significant change (Fig. 1D). These findings indicate the successful decellularization of the porcine skin tissue. Following the freeze-drying and pulverization processes, the decellularized porcine skin underwent pepsin digestion to generate a hydrogel precursor. Subsequently, the pH of the hydrogel precursor was modified, leading to a gradual solidification of the solution and ultimately yielding the porcine skin hydrogel (Fig. 1B, Additional file 1: Figure S1).

### The structure and porosity of Lev@PADM hydrogel

**Fig. 2:**
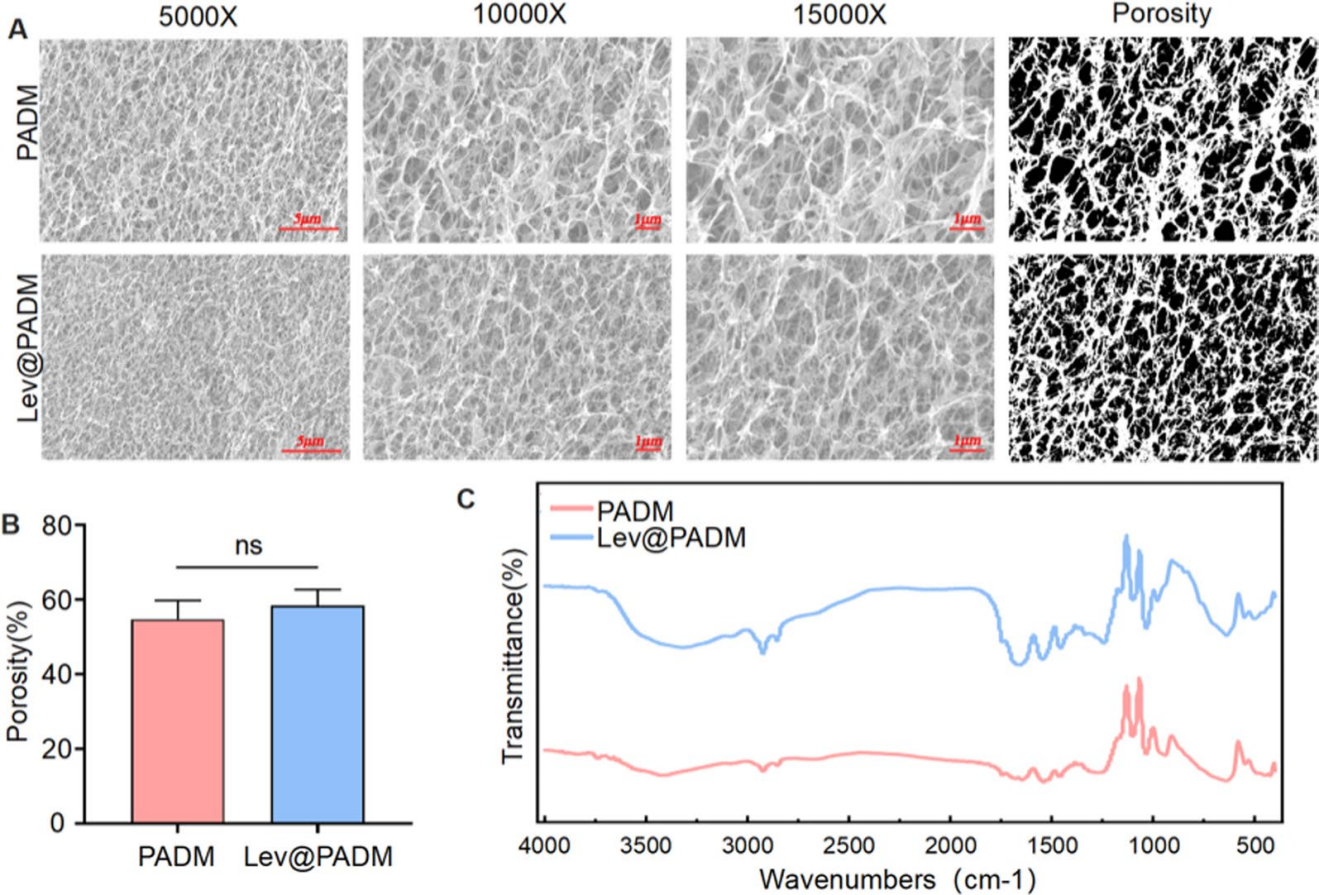
Porosity characteristics of PADM and Lev@PADM hydrogels. **A** Photographs of the hydrogels were captured at different magnifications of SEM (5000 ×, 10,000 ×, and 15,000 ×). Porosity images were computed at 10,000 × using Image J software. **B** The porosity of PADM hydrogel and Lev@PADM hydrogel was assessed. **C** Infrared spectral distribution of PADM hydrogel and Lev@PADM hydrogel under Fourier infrared spectroscopy

The scanning electron microscopy (SEM) images of porcine skin hydrogel and levofloxacin-hydrogel are depicted in Fig. 2A. The hydrogels were fabricated utilizing an equivalent mass of porcine skin powder, with the inclusion of levofloxacin at a concentration of 20 μg/ml. All two hydrogels exhibited fibrous network-like structures when observed under the microscope. Subsequent analysis using Image J software (Fig. 2B) revealed that the porosity of the two hydrogels was determined to be 53.53% and 59.21%, respectively, with a p-value exceeding 0.05. This finding suggests that the incorporation of the drug has an inconsequential impact on the porosity of the hydrogel. Fourier-transform infrared spectroscopy was employed for the analysis of porcine skin hydrogel, and levofloxacin-hydrogel. The obtained results (Fig. 2C) indicated the absence of substantial chemical bonding, implying the physical adsorption of drugs onto the hydrogel. This finding further substantiates the conclusion that the drugs did not impact the hydrogel’s porosity.

### Swelling performance, rheological characteristics, degradation rate, and drug release rate of Lev@PADM hydrogel

**Fig. 3:**
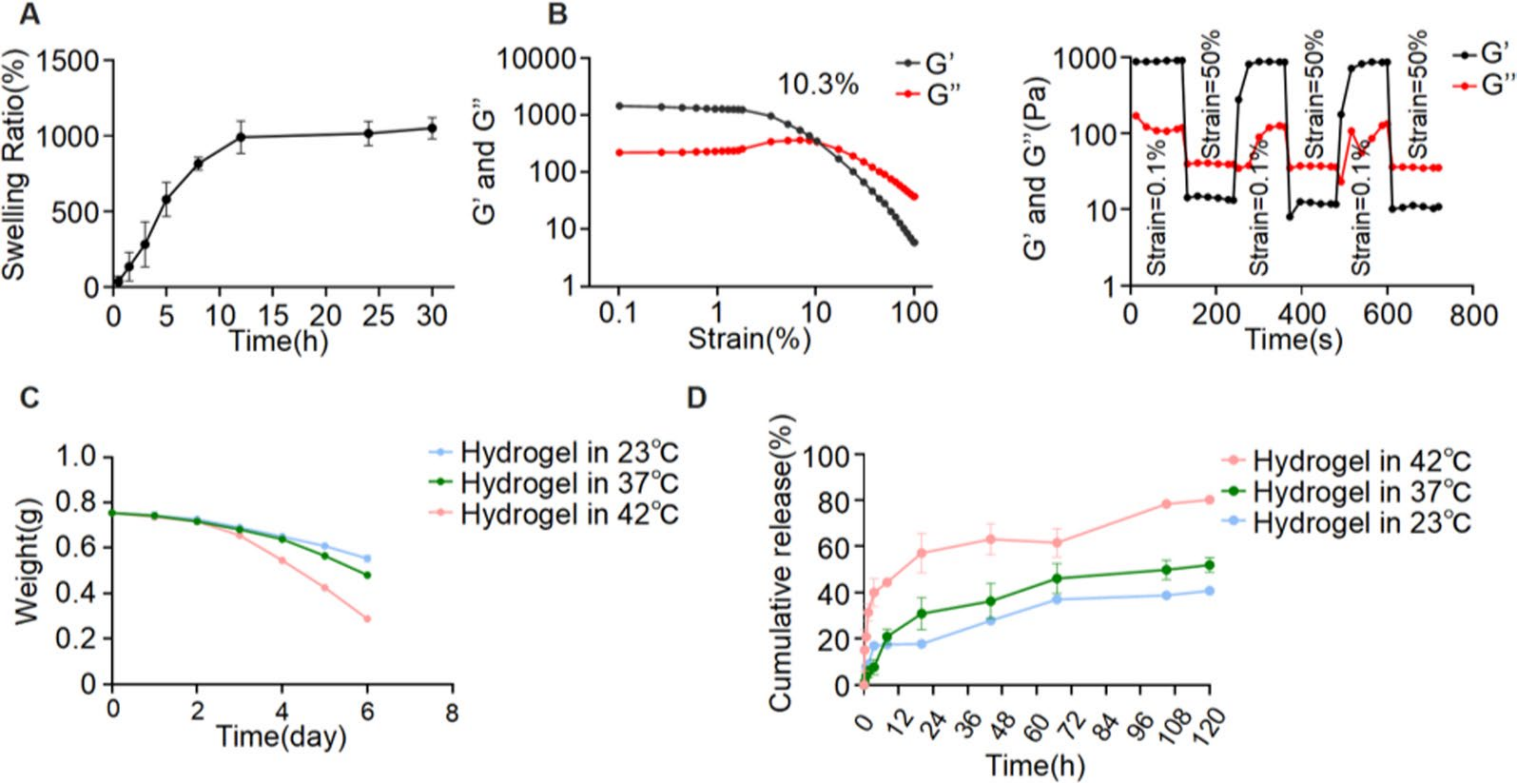
Swelling rate and resistance to deformation of decellularized porcine skin hydrogel. **A** PADM hydrogel swelling rate. **B** Variations in energy storage modulus (G’) and loss modulus (G’’) of PADM hydrogel in both strain-scan mode and time-scan mode. **C** Degradation of pigskin hydrogel over a span of 6 days at three different temperatures: 23 °C, 37 °C, and 42 °C. **D** Release of Levofloxacin from porcine skin hydrogel at the aforementioned temperatures

Research has shown that PADM hydrogels with a 50% porosity can achieve a swelling ratio of 1000% within 12 h (Fig. 3A), indicating that decellularized porcine dermis hydrogel can form a hydrophilic porous network structure in a short period of time. Levofloxacin is a water-soluble antibiotic, making it easily dispersed uniformly in the PADM hydrogel during the swelling process, as observed in previous studies.

The rheological characteristics of Lev@PADM hydrogel are depicted in Fig. 3B. Initially, during strain scanning, the energy storage modulus (G’) exhibits a gradual decline, while the loss modulus (G’’) demonstrates a gradual increase with increasing strain. These two moduli converge at a strain level of 10.3%. In the time-scan mode, the energy storage modulus (G’) and loss modulus (G’’) exhibit stability at a strain of 0.1%. However, a sudden increase in strain to 50% results in a decrease in the energy storage modulus (G’) and an increase in the loss modulus (G’’). Upon returning to a strain of 0.1%, the energy storage modulus (G’) is able to revert to its original level, and the loss modulus (G’’) also decreases to its initial level. These findings suggest that the Lev@PADM hydrogel possesses favorable resistance to deformation and can effectively maintain its morphology after being injected via a syringe. To further demonstrate that the hydrogel material does not cause urethral blockage after injection, we conducted a comparative analysis of bladder tissues treated with saline and Lev@PADM for 7 days. Results showed no significant increase in bladder tissue weight post-treatment, indicating no urethral obstruction caused by the hydrogel material (Additional file2: Figure S2).

The degradation of PADM hydrogel was examined at various temperatures (23, 37, and 42 °C) as depicted in Fig. 3C. It was observed that the degradation rate of PADM hydrogel escalated with rising temperature. Notably, no substantial disparity in degradation rates was observed among the three temperatures within the initial 48 h. However, after this period, the degradation rate of the hydrogel at 42 °C exhibited a gradual increase, resulting in the degradation of 0.455 g by day 6. In comparison, the degradation at 23 °C and 37 °C amounted to 0.203 g and 0.266 g, respectively. Through in vitro levofloxacin release experiments (Fig. 3D), we noticed that, under physiological temperature conditions, Lev@PADM releases a significant amount of levofloxacin within the initial 12 h. This release process occurs simultaneously with the swelling process of the hydrogel, indicating that the swelling process facilitates the diffusion of levofloxacin from the hydrogel into the surrounding aqueous medium. This suggests that levofloxacin can be rapidly released at the infection site, and this release process is temperature-dependent. It is worth noting that within 120 h at 37 °C, Lev@PADM releases less than 50% of the encapsulated levofloxacin, indicating that the slow release of levofloxacin begins after the swelling process of the hydrogel is completed. This characteristic of levofloxacin release aligns with the requirements for the application of Lev@PADM in the treatment of bladder infections.

### In vitro antibacterial activity

**Fig. 4:**
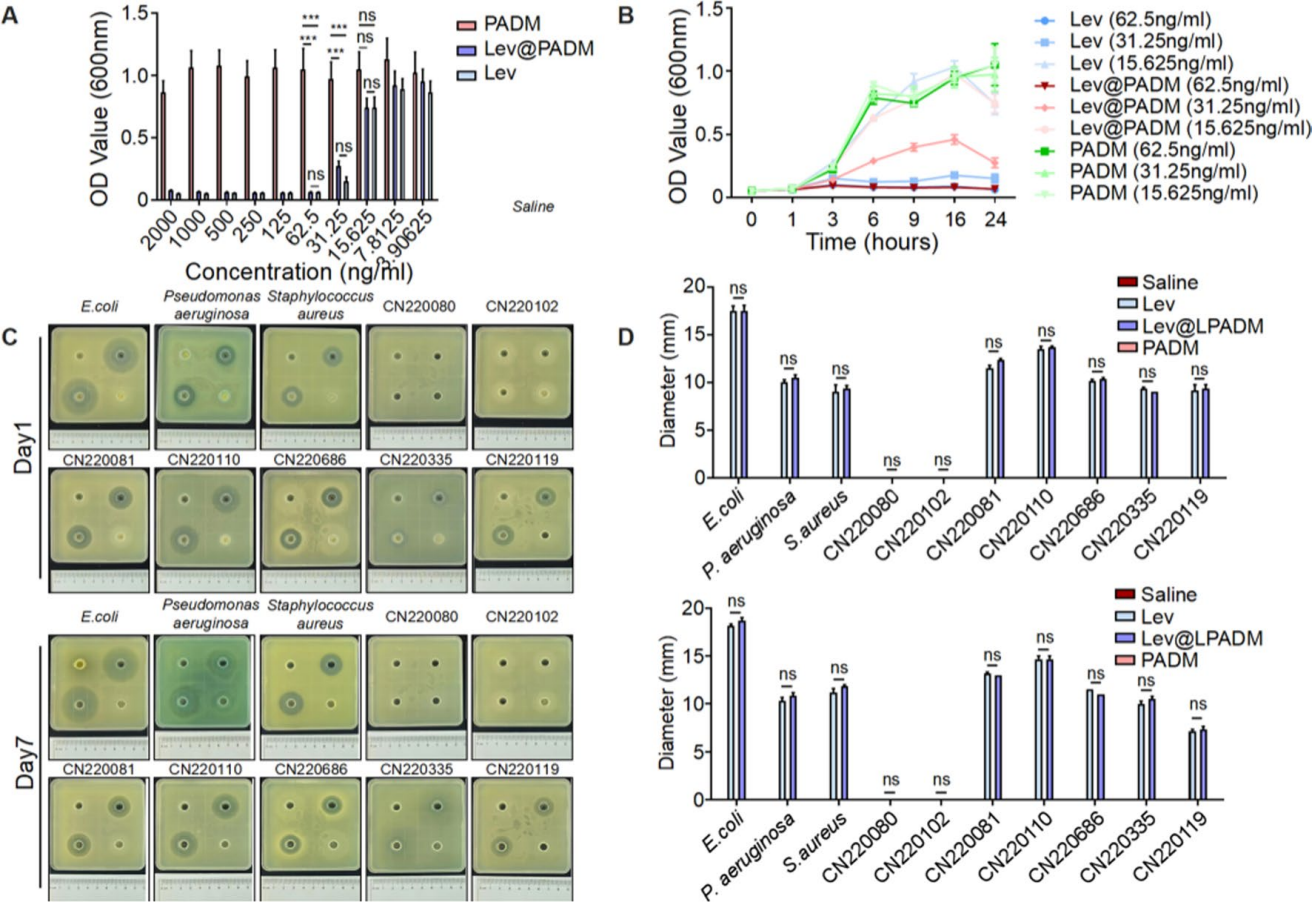
In vitro antimicrobial activity of Lev@PADM. **A** Inhibitory effects of PADM, Levofloxacin, and Lev@PADM on the growth of CFT073 at varying concentrations. **B** Growth curves of CFT073 under different treatment conditions. **C** Results of the antibacterial zone assay conducted on standard strains and clinical isolates cultured in PADM (low right), Levofloxacin (up right), blank medium (up left), and Lev@PADM (low left) for 1 and 7 days. **D** Statistical analysis of the results presented in **C**. (ns: no significant)

The concentration-dependent inhibitory effect of Lev@PADM on bacterial growth was investigated by co-culturing the standard strain of *E.coli* CFT073 with varying concentrations of Lev@PADM for a duration of 24 h. Both Lev@PADM and levofloxacin demonstrated significant inhibitory effects on *E.coli* when their concentrations reached 31.25 ng/ml, there was no statistically significant difference between the two groups (Fig. 4A). We conducted continuous OD600 measurements using three concentration groups: 62.5 ng/ml, 31.25 ng/ml, and 15.625 ng/ml. As a result, MIC of Lev@PADM and levofloxacin both ranged from 31.25 to 15.625 ng/ml. Notably, after 6 h of cultivation at a concentration of 31.25 ng/ml, both Lev@PADM and levofloxacin demonstrated significant antibacterial properties (Fig. 4B). In order to investigate the efficacy of levofloxacin on different strains of *Escherichia coli*, we conducted bacteriostatic experiment on seven clinical isolates. The results indicated that both Lev@PADM and levofloxacin exhibited antibacterial activity against susceptible strains of *Escherichia coli* (Fig. 4C, D). Our investigation also examined the inhibitory effects of Lev@PADM on other standard strains of *Staphylococcus aureus* and *Pseudomonas aeruginosa*, which are commonly associated with urinary tract infections. In accordance with the findings from the bacteriostatic experiment conducted on *Escherichia coli*, both Lev@PADM and levofloxacin exhibited discernible bacteriostatic circles. Moreover, there was no statistically significant disparity observed in the diameters of the bacteriostatic circles between the two groups (Fig. 4C, D). These results provide additional evidence that the synthesis process of Lev@PADM does not impede the inhibitory efficacy of levofloxacin against diverse pathogenic bacteria responsible for urinary tract infections. It should be noted that while Lev@PADM significantly enhances the local concentration of levofloxacin through targeted delivery, thereby avoiding the selection process of drug-resistant strains at low concentrations of the antibiotic, it does not have an effect on strains that have already developed resistance (Fig. 4C, strains CN220102 and CN220080). Therefore, when utilizing Lev@PADM for intervention in urinary tract infections, it is still necessary to conduct antimicrobial susceptibility testing.

### In vitro safety evaluation

**Fig. 5:**
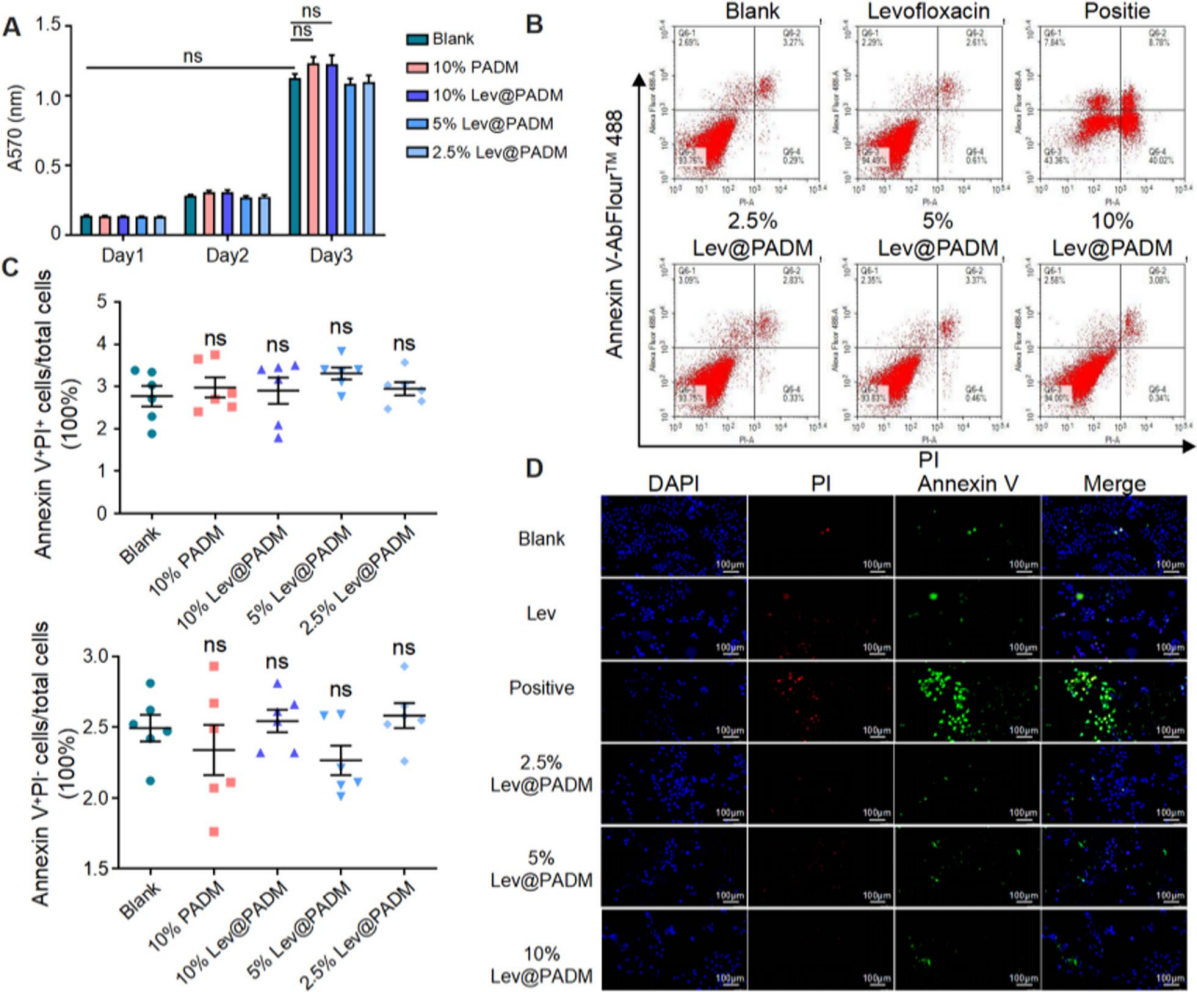
In vitro safety evaluation of Lev@PADM. **A** Inhibitory effects of PADM, Levofloxacin, and Lev@PADM on the growth of CFT073 at varying concentrations. **B** Results of flow cytometry detection of apoptosis in the 5637 cell line under treatment conditions of PADM, Levofloxacin, and Lev@PADM. **C** Statistical analysis of the flow cytometry results from **B**. **D** Fluorescence dual staining results of PI and Annexin V in the adherent 5637 cell line after 24 h of treatment. (ns: no significant)

Prior to achieving the objective of optimized diagnosis or treatment of infectious diseases through the delivery of drugs, genes, or proteins using materials as carriers, it is paramount to prioritize the biocompatibility and safety of these materials [38]. The objective of this study was to investigate the efficacy of intravesical injection of Lev@PADM for the treatment of bacterial cystitis. It is imperative to ensure that Lev@PADM exhibits good biocompatibility, enabling direct interaction with epithelial cells, as this is crucial for preserving the integrity of the bladder epithelial barrier [39, 40]. The Lev@PADM hydrogel was co-cultured with the 5637 cell line for a duration of 48 h, and the cytotoxicity of the Lev@PADM hydrogel on bladder epithelial cells was assessed using the PI/Annexin V staining assay. The findings revealed that, in comparison to the control group, there was no significant increase observed in early or late apoptosis of the 5637 cells co-cultured with Lev@PADM hydrogel at concentrations of 10%, 5%, and 2.5% (Fig. 5B–D). Furthermore, the MTT assay was conducted on cells that were continuously co-cultured with Lev@PADM hydrogel to further investigate its impact on cell proliferation. The results of the study revealed a significant increase in the number of 5637 cells after three days of normal culture. Co-culturing with PADM (10%) and Lev@PADM hydrogel (10%, 5%, 2.5%) did not result in a significant decrease in cell proliferation compared to the blank control group (p > 0.05) (Fig. 5A). Within the concentration range of 2.5–10%, PADM hydrogel does not hinder the proliferation of bladder epithelial cell lines.

To further address the effects on bladder tissue, we compared the histological features of bladder tissues treated with Lev@PADM, saline, levofloxacin, and PADM. The HE staining results demonstrated that, similar to the saline and levofloxacin groups, the Lev@PADM group did not induce immune cell infiltration or significant changes in the bladder epithelial structure (Additional file 3: Figure S3A). Additionally, the Tunel assay results showed that Lev@PADM treatment did not significantly increase bladder epithelial cell apoptosis (Additional file 3: Figure S3A, B, p > 0.05).

Based on these findings, it can be concluded that Lev@PADM hydrogel not only effectively and rapidly eradicates bacteria in vitro but also exhibits favorable biocompatibility.

### In vivo antibacterial test

In this study, we conducted a novel intervention on bacterial cystitis caused by *E.coli* using Lev@PADM hydrogel via bladder instillation. Following established research methods [41], we established a model of bacterial cystitis and treated four groups of model mice with saline, levofloxacin, PADM, or Lev@PADM intervention on the third day of initial infection. The results demonstrated a significant reduction in colony counts in the urine of the levofloxacin and Lev@PADM treatment groups on the second day. The findings indicate that both levofloxacin and Lev@PADM were effective in reducing the colony counts of *E.coli* in the bladder, as evidenced by a significant reduction in urine colony counts on the second day after intervention. However, on the seventh day after infection, the bacteriostatic effect of levofloxacin diminished, whereas the Lev@PADM group continued to exhibit significantly lower bacterial counts in urine compared to the saline group. This suggests that Lev@PADM has a more prolonged bacteriostatic effect in comparison to levofloxacin.

**Fig. 6:**
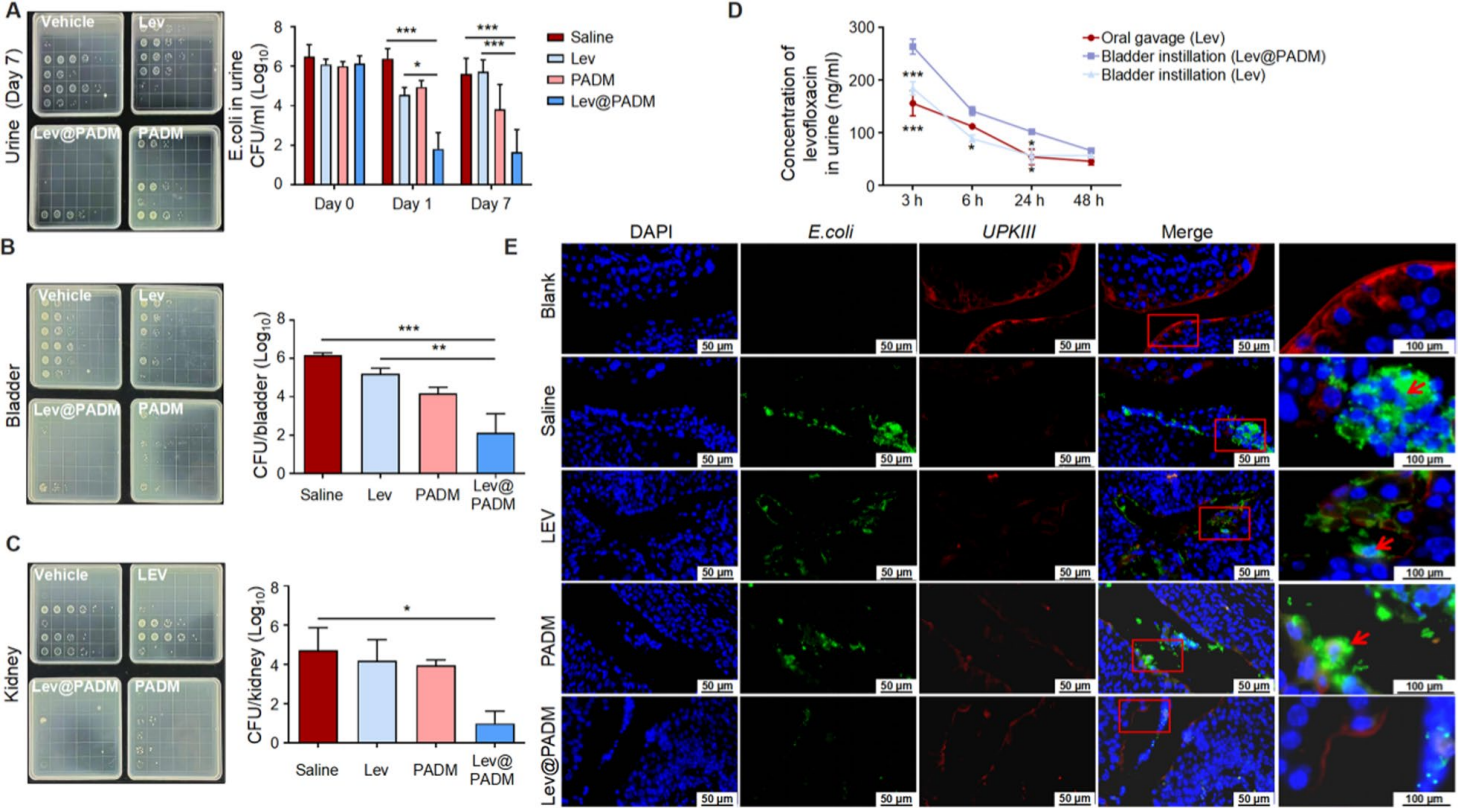
Long-lasting inhibition of bacterial cystitis by intravesical injection of Lev@PADM. **A**–**C** Bacterial colony counts in urine, bladder, and kidney following treatment with saline (up-left), Levofloxacin (up-right), PADM (down-right), and Lev@PADM (down-left) in a bacterial cystitis model. **D** Time-dependent changes in levofloxacin concentration in mouse urine after a single administration. **E** Fluorescence co-localization of *E. coli* and UPKIII in the bladder tissue on the 7th day post-treatment. (*p < 0.05; **p < 0.01; ***p < 0.001. Scale bar: 50 μm. Red arrows indicate positively stained cells with intracellular bacteria)

In addition to intracellular invasion of epithelial cells leading to recurrent urinary tract infections, *E.coli* can also ascend through the ureters to the kidneys, causing more severe pyelonephritis [42, 43]. By counting the colonies in bladder and kidney tissues, we found that the Lev@PADM group significantly reduced the number of bacteria in the bladder tissue compared to the saline and levofloxacin groups (Fig. 6B). Similarly, Lev@PADM also significantly reduced the invasion of *E.coli* into the kidneys (Fig. 6C), indicating that Lev@PADM is better at inhibiting the invasion of *E.coli* into the entire urinary system compared to levofloxacin. Co-localization of *E.coli* and UPKIII in bladder tissue was performed, and the results showed that the expression of UPKIII was greatly reduced in all four treatment groups, indicating the occurrence of epithelial cell shedding during bacterial infection. However, a large number of *E.coli* and intracellular bacteria were only present in the bladders of the levofloxacin, saline, and PADM groups, while Lev@PADM significantly reduced the adhesion of *E.coli* to epithelial cells and the formation of intracellular bacteria through bladder instillation (Fig. 6E). This result suggests that the bladder instillation intervention with Lev@PADM has the potential therapeutic effect of inhibiting the recurrent occurrence of urinary tract infections.

Previous research has demonstrated the favorable adhesive properties of PADM hydrogel, which enables the formation of a tightly adhered physical barrier on the contact surface [23, 44, 45]. Additionally, our prior in vitro investigations have substantiated the sustained release attributes of Lev@PADM. Consequently, we conducted a comparative analysis of the alterations in levofloxacin concentration in urine over a 48-h period following treatment with three distinct approaches: oral administration of levofloxacin, bladder instillation of levofloxacin, and bladder instillation of Lev@PADM, all administered at a dosage of 20 μg/ml (Fig. 6D). The results showed that starting from three hours after administration, the concentration of levofloxacin in the urine of the Lev@PADM bladder instillation group was significantly higher than that of the levofloxacin oral administration group and the levofloxacin bladder instillation group. Furthermore, 24 h after administration, the levofloxacin concentration in the urine of the Lev@PADM group remained above the minimum inhibitory concentration (greater than 31.25 ng/ml) and was significantly higher than that of the levofloxacin oral administration group and the levofloxacin bladder instillation group. Hence, the utilization of Lev@PADM hydrogel has demonstrated a substantial extension in the duration of levofloxacin’s efficacy within the bladder, potentially attributing to its capacity to impede the persistence of pathogenic bacteria in bladder.

### Lev@PADM alleviates the excessive immune response following bladder infection by inhibiting the activation of the NF-κB signaling pathway

**Fig. 7:**
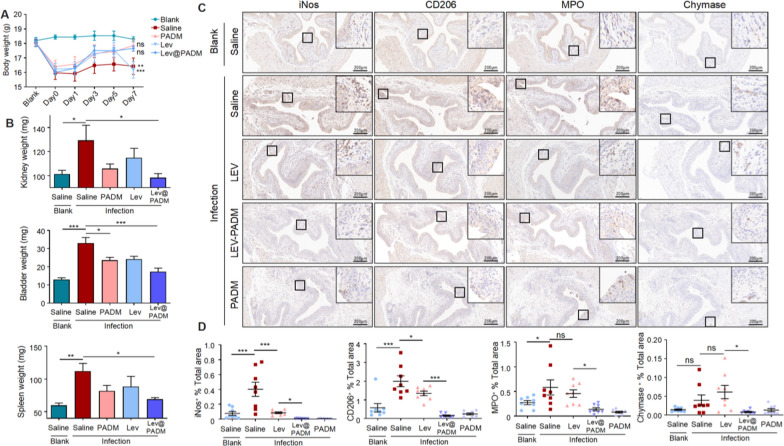
The impact of Lev@PADM on the innate immunity in cases of bacterial cystitis. **A** Variations in body weight prior to and following the administration of saline, Levofloxacin, PADM, and Lev@PADM in a bacterial cystitis model. **B** Weight of kidneys, bladder, and spleen in mice belonging to different treatment groups after 7 days of infection. **C** Expression of iNOS, CD206, MPO, and Chymase in bladder tissue on the 7th day post- treatment. **D** Optical density analysis of positive cells in **C**. (*p < 0.05; **p < 0.01; ***p < 0.001; ns: no significant. Scale bar: 200 μm. Black boxes indicate representative areas of positive protein expression)

The role of the bladder immune response is crucial in determining the outcome of urinary tract infections (UTIs). Specifically, the innate immune system, comprising resident and recruited cells, identifies pathogens through pattern recognition receptors like TLR2, TLR4, TLR5, and TLR11 [46–48], resulting in prompt and vigorous pro-inflammatory reactions. In recent years, there has been a significant utilization of immune-modulatory compounds, such as polydopamine (PDA), polyphenols [49], amlodipine [50], polyphosphazene macromolecular adjuvants [51], selenium nanoparticles (SeNPs) [52], and more, in material synthesis. The combined application of these compounds undoubtedly enhances the functionality of materials and expands their potential applications. To further investigate the impact of Lev@PADM’s long-lasting antimicrobial effect on the innate immune response, we first measured the body weight and organ weight of mice in each group before and after infection. Results showed a significant decrease in body weight and a notable augmentation in bladder, spleen, and kidney weight. Solely the mice treated with Lev@PADM demonstrated a substantial decrease in organ weight, subsequently restoring them to their baseline levels (Fig. 7A, B). This observation suggests that the administration of Lev@PADM effectively mitigated the enlargement of the bladder, kidney, and spleen induced by bacterial infection. Immunohistochemistry (IHC) staining was performed for neutrophils, M1 macrophages, M2 macrophages, and mast cells, which are prominent innate immune cells in the tissue (Fig. 7C, D). The findings revealed a significant increase in the quantities of neutrophils, M1 macrophages, and M2 macrophages in the bladder tissue of infected mice after a duration of seven days, potentially linked to the persistent presence of *E.coli* in the bladder (Fig. 6B). The bladder treated with Lev@PADM exhibited a significant decrease in the quantities of neutrophils, M1 macrophages, M2 macrophages, and mast cells compared to the levofloxacin-treated group. This suggests that the Lev@PADM hydrogel not only extends the antimicrobial efficacy of levofloxacin and diminishes bacterial retention in the bladder, but also suppresses excessive activation of the innate immune response.

In order to further investigate the mechanism by which Lev@PADM inhibits innate immunity, we performed Western blot (WB) analysis on treated bladder tissue to examine the levels of TLR4, p-P65, p-IκB, TNFα, and IL-1β (Additional file 4: Figure S4A, B). Extensive research has shown that *E. coli* can activate the NF-κB signaling pathway by binding to TLR4, promoting the phosphorylation and nuclear translocation of P65 (Additional file 5: Figure S5A, B), and subsequently transcribing TNFα and IL-1β to promote immune cell infiltration and activation. However, excessive immune response can also lead to barrier disruption, thereby promoting *E. coli* invasion of the urinary tract. Therefore, mitigating NF-κB signaling activation, while controlling bacterial infection, is beneficial for the restoration of the bladder epithelial barrier. Our results revealed a significant upregulation of the NF-κB signaling pathway in bladder tissues after infection. Treatment with levofloxacin inhibited NF-κB activation, and compared to levofloxacin, Lev@PADM further suppressed NF-κB activation. This suggests that Lev@PADM treatment may potentially reduce NF-κB activation, decrease the expression of inflammatory factors TNF-α and IL-1β, and subsequently diminish immune cell infiltration, which may be the main reason for Lev@PADM’s reduction in immune cell infiltration. Consequently, this advantageous outcome contributes to the preservation of the bladder epithelial barrier’s integrity and facilitates the recuperation from urinary tract infections.

### Maintenance of gut microbiota structure

The perturbation of the gut microbiota resulting from the administration of antimicrobial agents is linked to dysregulation of the host immune system, thereby heightening vulnerability to diverse bacterial infections [53, 54]. Prior investigations have unequivocally established that the modification of the gut microbiota composition brought about by levofloxacin intensifies the inflammatory reactions of the host [55]. This implies that mitigating the influence of levofloxacin on the gut microbiota holds considerable significance in the management of urinary tract infections.

**Fig. 8:**
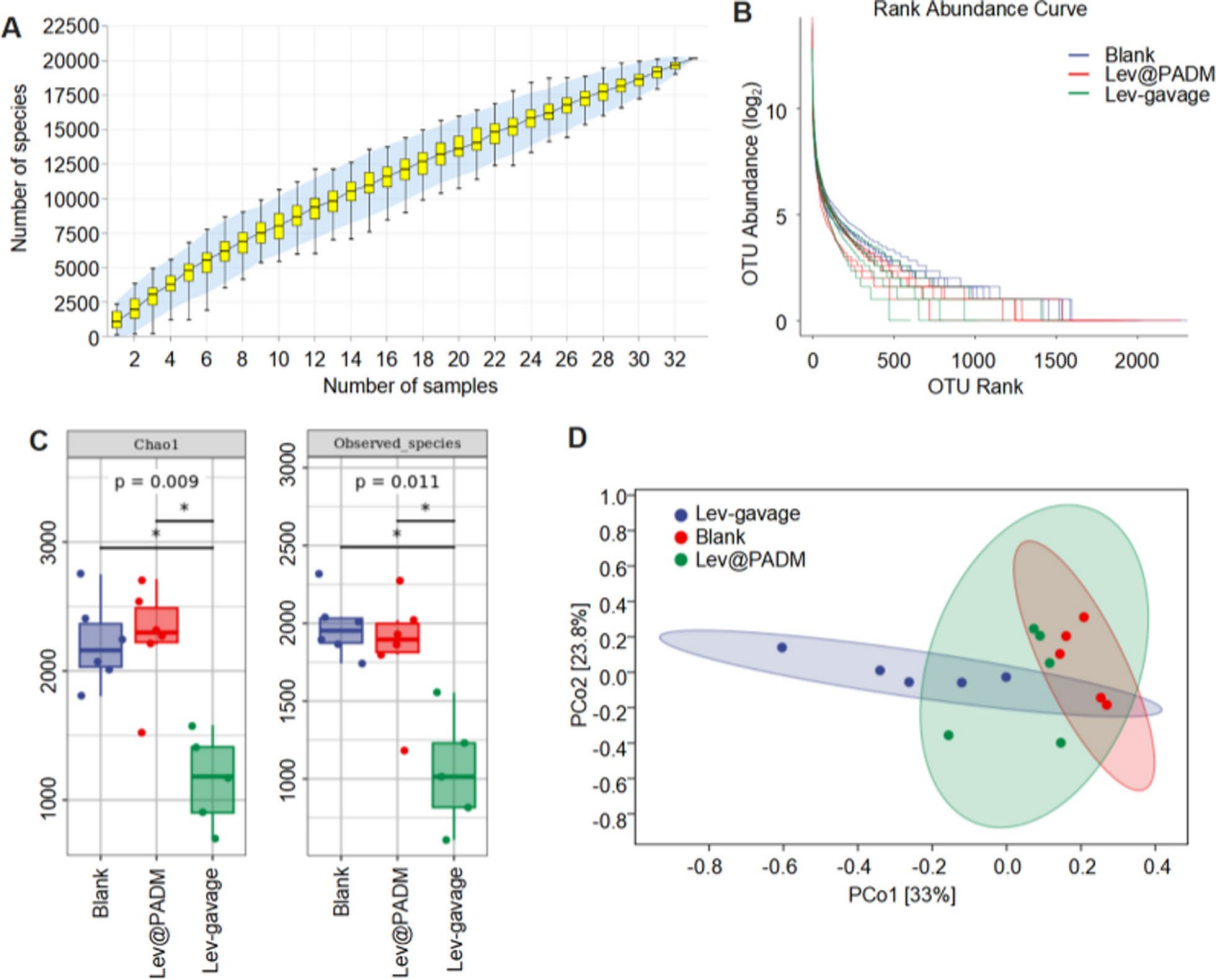
Intravesical infusion of Lev@PADM effectively prevents disruption of the gut microbiota. **A** Accumulation curve of OTU species. **B** Rank-abundance curve. **C** Alpha diversity analysis of gut microbiota. **D** PCA analysis of gut microbiota composition in different groups. (*p < 0.05.)

In this study, we conducted 16S rRNA sequencing on fecal samples obtained from three groups of mice: the control group, the levofloxacin oral gavage group, and the Lev@PADM bladder instillation group. To identify species and genes, Operational Taxonomic Units (OTUs) were clustered at a 97% similarity level. The OTU species accumulation curve reached a plateau as the sample size increased, suggesting that the sample size was sufficient for the experimental requirements (see Fig. 1A). Additionally, the rank-abundance curve indicated a wider range of species abundance in the Lev@PADM group compared to the levofloxacin group, suggesting a higher species abundance in the Lev@PADM group. Furthermore, the α-diversity analysis of the gut microbiota, specifically Chao1 and Observed-species metrics, revealed no statistically significant disparities in species abundance between the Lev@PADM group and the control group. Conversely, the levofloxacin oral gavage group displayed noteworthy alterations in species abundance when compared to the control group, as depicted in Fig. 7C. The proximity of scatter points in the PCA plot indicates minimal variations within the intra-group samples, thereby implying the experiment’s satisfactory repeatability and reliability. The samples obtained from the group administered with levofloxacin orally and the control group were observed to be distinct from each other, implying that oral gavage of levofloxacin resulted in alterations in the composition of gut microbiota. Notably, the samples acquired from the Lev@PADM bladder instillation group exhibited a closer resemblance to the control group. This observation indicates that bladder instillation of Lev@PADM offers superior preservation of the structure of gut microbiota in comparison to oral administration of levofloxacin, thereby emphasizing the potential of bladder instillation with Lev@PADM as a therapeutic approach for urinary system infections (Fig. 8).

## Discussion

We conducted a comprehensive investigation into the physical properties and biological activity of Lev@PADM hydrogel, which was introduced for the first time in the treatment of bacterial cystitis. Our findings highlight three advantages of utilizing Lev@PADM hydrogel for the treatment of bacterial cystitis. Firstly, the hydrogel exhibits sustained release of levofloxacin and remarkable resistance to deformation. This feature allows for targeted elevation of local levofloxacin concentration at the site of infection through bladder injection, thereby avoiding the hepatotoxic, nephrotoxic, and neurotoxic side effects that may arise from high systemic drug concentrations. Furthermore, the Lev@PADM hydrogel demonstrates favorable biocompatibility and anti-inflammatory characteristics. Consequently, in conjunction with its antimicrobial properties, it effectively sustains the integrity of the bladder epithelium, thereby positively impacting the prognosis of bladder infections. Lastly, when compared to the traditional oral administration of levofloxacin, the intravesical administration of Lev@PADM hydrogel preserves the composition of the indigenous gut microbiota. This aspect holds considerable significance in terms of maintaining the host’s immune function and preventing the recurrence of urinary tract infections.

Hydrogel materials, through the encapsulation of antibiotics, metal nanoparticles, and other antimicrobial agents, have been widely utilized in the treatment of bacterial infections [56]. According to reports, HA-PAR hydrogel has demonstrated persistent antibacterial activity in both in *vitro* and in *vivo* experiments by releasing polyarginine over an extended period [19]. Similarly, P(AA_10_-co-HEMA_10_)_Gen_ hydrogel exhibits a pH-dependent sustained release of gentamicin for the treatment of skin infections [57]. These studies suggest that the use of antibacterial drug-loaded hydrogels may offer a time advantage for the treatment of bladder infections. Despite the existence of extensive research studies on the utilization of hydrogel systems for intravesical drug delivery [58, 59], the implementation of Lev@PADM in the management of bacterial cystitis presents an innovative intervention. Hydrogels possess the capacity to mitigate drug dilution and confer adhesive or floating characteristics to loaded substances, thereby prolonging the drug’s efficacy within the bladder [60, 61]. A study documented the development of a mucoadhesive hydrogel composed of high molecular weight chitosan and β-glycerophosphate, which exhibited enhanced resistance to urine washout when evaluated ex vivo using porcine urinary bladder, thus presenting a potential treatment option for bladder cancer [62]. In a separate study, floating hydrogels comprised of Poloxamer 407 (P407) were investigated for their efficacy in managing bladder-related diseases [63]. The hydrogel demonstrated prolonged residence time and continuous drug release for up to 10 h when tested in vivo using a rabbit acute bladder injury model. In our investigation, we employed decellularized porcine dermis hydrogel as the carrier for Levofloxacin. This bio-matrix hydrogel endowed Lev@PADM with adhesive properties and gradual release attributes, enabling it to adhere to bladder epithelial cells and furnish a biologically benign *E.coli* protective barrier that emulates the inherent mucosal surface. Consequently, PADM can collaborate with therapeutic drugs, particularly in instances where the mucosal barrier is compromised.

*E.coli* is the primary causative bacterium responsible for bacterial cystitis. The lipopolysaccharides (LPS) present on its surface bind to the TLR4 receptors located on the epithelial cells of the bladder, thereby initiating the activation of IKK and facilitating the translocation of NF-κB into the nucleus. This sequential process ultimately leads to the regulation of various pro-inflammatory cytokines, including IL-1β, TNF-α, and IL-6 [9, 64–67]. Consequently, the activation of Ly6C − macrophages and mast cells occurs, while neutrophils and Ly6C + macrophages are recruited to eliminate uropathogenic *E.coli* (UPEC) that has invaded the bladder epithelial cells [68]. However, an overabundance of inflammation can also lead to compromised tight junctions, heightened barrier permeability, and deficiencies in umbrella cells. The absence of fully developed epithelial cell coverage renders the basal epithelial cells more vulnerable to UPEC adhesion, biofilm formation, and even intracellular bacterial colonization, resulting in the persistence of bacterial cystitis [69]. Our research findings have shown that, in comparison to levofloxacin, Lev@PADM consistently inhibits the proliferation of *E.coli* within the bladder and significantly diminishes intracellular bacterial production in bladder tissue. The results of our study indicate that Lev@PADM exhibits prolonged inhibition of *E.coli* proliferation in the bladder and effectively reduces intracellular bacterial production in bladder tissue, when compared to levofloxacin. These effects can potentially be attributed to the adhesive characteristics of Lev@PADM to the bladder epithelial barrier, its sustained release of levofloxacin, and its ability to suppress excessive immune responses within the bladder.

The intestinal microbiota is widely recognized as the second genome of the human body [70], exerting a pivotal influence on the regulation of host immunity and gastrointestinal function [71]. Previous studies have consistently indicated that the administration of levofloxacin can disturb the equilibrium of the intestinal microbiota, thereby inducing antibiotic-associated inflammation within the gastrointestinal tract [55]. Moreover, previous research conducted by our team and other scholars has provided evidence that the depletion of the intestinal microbiota resulting from the administration of antimicrobial drugs substantially elevates the likelihood of bacterial infectious diseases [53, 54, 72–74]. This correlation is closely linked to the reduction of immunoregulatory metabolites derived from the intestinal microbiota. Consequently, it is imperative to closely monitor the stable composition of the intestinal microbiota when employing levofloxacin for the management of urinary tract infections, as it exerts a substantial influence on the prognosis of the infection. In this study, we compared the effects of oral levofloxacin and bladder instillation of Lev@PADM on the intestinal microbiota structure in mice. We found that bladder instillation of Lev@PADM avoided the disruption of the intestinal microbiota caused by levofloxacin. This finding suggests that Lev@PADM, as a biomaterial, holds great potential for the treatment of urinary tract infections.

## Conclusion

The immobilization of levofloxacin onto PADM hydrogel through physical adsorption has led to the development of Lev@PADM hydrogel composite, which exhibits controlled drug release properties. This innovative approach offers several advantages for the administration of levofloxacin, as the hydrogel composite possesses excellent resistance to deformation, allowing for its injection into the bladder. Importantly, the synthesis process employed in creating Lev@PADM does not compromise the inherent antibacterial activity of levofloxacin. In fact, our study demonstrates that Lev@PADM exhibits extended antibacterial efficacy compared to levofloxacin alone. This enhanced effectiveness is complemented by its ability to modulate the immune response, thereby aiding in the restoration of the bladder epithelial barrier and preventing bacterial reattachment in urine following infection. Moreover, we have observed that Lev@PADM exerts a reduced impact on the intestinal microbiota compared to levofloxacin alone. This is a significant finding, as it suggests that Lev@PADM could potentially minimize the disruption of the gut microbiome, which is crucial for maintaining overall health. Based on these compelling attributes, Lev@PADM has emerged as a highly promising biomaterial for the management of urinary tract infections. Its slow drug release characteristics, extended antibacterial efficacy, immune regulatory capabilities, and reduced impact on the intestinal microbiota make it a valuable candidate for further exploration and development in the field of urinary tract infection treatment. However, it is important to acknowledge the limitations of our study. Although our experiments demonstrate the potential of Lev@PADM, further research is needed to optimize its formulation, dosage, and long-term safety profile. Additionally, the complex dispersion characteristics of Lev@PADM, compared to levofloxacin alone, warrant careful consideration in future studies.

In conclusion, the immobilization of levofloxacin onto PADM hydrogel to create Lev@PADM represents an innovative approach with significant potential for the management of urinary tract infections. The extended antibacterial efficacy, immune regulatory capabilities, and reduced impact on the intestinal microbiota make Lev@PADM a promising biomaterial for further investigation and development.

## Materials and methods

### Preparation of Lev@PADM hydrogel

Fresh pig skins were procured from slaughterhouses and subsequently processed by removing soft tissues such as muscle and fascia. These tissues were then sliced into thin sections measuring 2–3 mm in thickness. The slices were placed in beakers and subjected to a three-hour rinsing period at room temperature (25 °C) using sterile water with continuous agitation. Following this, the slices were frozen and thawed for three cycles, with temperatures ranging from − 80 °C to 37 °C. The resulting thin slices of pork skin were immersed in a 1% Triton X-100 (v/v) solution, which was refreshed every 24 h. After a 24-h period, the slices were transferred into a 0.1% Ethylene diamine tetraacetic acid (EDTA) solution and stirred for an additional 24 h. The slices underwent an overnight rinsing process with a substantial quantity of phosphate buffered saline (PBS) to eliminate any remaining chemicals. Subsequently, the treated flakes were subjected to lyophilization using a freeze dryer, and upon obtaining the lyophilized flakes, they were further pulverized into a powder using a grinder. A concentration of 12 mg/ml was introduced into a solution of HCl with a pH range of 2 to 3, which also contained pepsin at a mass ratio of 1:2 with PADM.

powder. The mixed suspension was subjected to continuous stirring for a duration of 24 h at ambient temperature until it achieved homogeneity, resulting in the formation of a flowable pre-gel solution. Subsequently, levofloxacin was incorporated into the pre-gel solution in accordance with the pre-gel volume, thereby obtaining a hydrogel loaded with the levofloxacin. The pre-gel solution was neutralized using a pre-cooled 0.1 M NaOH solution, and the osmotic pressure of the neutralized pre-gel solution was adjusted utilizing PBS. Following the aforementioned procedures, the pre-gel solution was loaded into a syringe, injected into the mold, and left undisturbed at ambient temperature (25 °C) for a duration of 5 min.

### Characterisation of hydrogels

The effect of decellularisation was evaluated through the utilization of HE and DAPI staining techniques, which allowed for the observation of decellularisation of the porcine epidermal matrix under a microscope. The hydrogels were subjected to fixation in a 2.5% glutaraldehyde solution at room temperature for a duration exceeding one hour. Subsequently, the samples underwent a series of washing steps, involving three repetitions of a 10-min wash with deionized water. Following this, the samples were subjected to dehydration using ethanol solutions with varying concentrations, namely 30%, 50%, 70%, 80%, 90%, and 95%, with each concentration being applied for a duration of 15 min. Finally, the samples were treated with a 100% ethanol solution and soaked. The specimens were subjected to dehydration using liquid carbon dioxide in a critical point desiccator. Subsequently, the scanning electron microscope (JSM-6360LV, Japan) was employed to examine the structural and surface characteristics of the PADM hydrogel and Lev-PADM hydrogel after sufficient dehydration treatment. The porosity of the cross-sections of both hydrogels was determined by capturing SEM images at a magnification of 10000X, which were then analyzed using Image J software (National Institutes of Health, USA).

### Determination of DNA content and collagen content

Small slices of pork skin were cut and a sample weighing 5–25 mg was added to a test tube. After tissue lysis and washing, a DNA extraction kit (TaKaRa, Japan) was used to obtain a purified DNA solution. Subsequently, 100 μl of the solution was added to a 96-well plate, and the OD values were measured at 260 nm using a spectrophotometer to calculate the DNA content. The determination of collagen involved measuring the hydroxyproline content using the chloramine assay, with a hydroxyproline (HYP) to collagen ratio of 1:7.6954. The hydroxyproline concentration standard curve was initially obtained through spectrophotometry using a hydroxyproline ELISA kit. The methodology involved a concise description of the sample’s hydrolysis in 38% concentrated hydrochloric acid at 110 °C for a duration of 18 h, followed by pH adjustment to a range of 6–8. Subsequently, 100 μl of the resulting solution was added to a 96-well plate, and the collagen content was determined by calculating the absorbance at a wavelength of 560 nm using a spectrophotometer.

### Fourier-infrared spectroscopy

The infrared spectra of PADM hydrogel, and Lev@PADM hydrogel were analyzed using Fourier-infrared spectroscopy. The examination was conducted through the potassium bromide method, utilizing a Fourier infrared spectrometer (Nicolet Nexus, Thermo, USA) that operates within the 400–4000 cm^−1^ range.

### Rheological analysis

Using a rotational rheometer (MCR302, Anton Paar, Canada), the PADM hydrogel was placed on a parallel plate at 37 °C. The strain was set from 0.1% to 100%, and the frequency was set to 10 rad/s for a total of 10 min. Each cycle lasted 2 min.

### Swelling properties, in vitro degradation

Swelling rate: A 5 ml centrifugal tube was filled with 0.5 ml of hydrogel precursor solution. The gelatinized and freeze-dried hydrogel was then incubated by adding equal volumes of PBS. Several times during the incubation period, the weight of each sample was measured, and the percentage swelling of the gel was determined.

Degradation rate: An amount of pig skin hydrogel precursor is placed into a 15 ml centrifugal tube, after gelation, 10 ml PBS solution is added, and the centrifugal tube is placed at 23 °C, 37 °C, and 42 °C. After a certain period to suck out the liquid in the centrifugal tube, weigh the sample.

### Release of levofloxacin

In order to investigate the release of Levofloxacin from Lev@PADM hydrogel under varying conditions, standardized UV absorbance curves were subsequently established to calibrate the measurements. The release of Levofloxacin from the Lev@PADM hydrogel was then evaluated at different temperatures. Specifically, 500 μl of Lev@PADM with a drug concentration of 20 μg/ml was placed in centrifuge tubes containing 500 μl of phosphate buffer and incubated at temperatures of 25, 37, or 42 °C. Subsequently, 100 μl of the resulting liquid was analyzed and.added back to the centrifuge tube. Repeat this procedure until the OD of the solution stops increasing.

### Cell culture experiment

To perform the cell culture experiment, the Lev@PADM hydrogel was diluted with 1640 medium to obtain concentrations of 10%, 5%, and 2.5% in the overall culture system. These culture systems were then utilized for the cultivation of the 5637 cell line over a period of three consecutive days. The MTT assay kit was employed to assess cell proliferation and growth status during the culture process. Furthermore, the PI/Annexin V assay kit was utilized to detect cell apoptosis.

### Continuous antibacterial test

To conduct the continuous antibacterial test, the Lev@PADM hydrogel was diluted with MHB medium, with a gradual decrease in levofloxacin concentration from 2000 ng/ml to 3.90625 ng/ml in a gradient fashion. The resulting medium was then utilized for the cultivation of the CFT073 strain for a duration of 24 h. Throughout the cultivation process, the OD600 value of the CFT073 strain was continuously monitored using a spectrophotometer.

Bacteriostatic circles were measured using a standardized method. Specifically, logarithmic phase cultures of *Escherichia coli*, *Pseudomonas aeruginosa*, and *Staphylococcus aureus* were mixed with MHA medium at a density of 10^5^ CFU/ml. This mixture was then poured into culture plates containing pre-placed Oxford cups. Once the medium solidified, 200 μl of sterile saline, levofloxacin, Lev@PADM hydrogel, and PADM were added to the respective Oxford cups. The plates were subsequently incubated for a period of 7 days. Following the incubation period, the diameters of the bacteriostatic circles were measured.

### Mouse models of *E.coli*-induced bacterial cystitis

Seven-to-eight-week-old Female C57BL/6 mice were purchased from GemPharmatech Co. Ltd. (Nanjing, China). Mice were allowed to recover from the stress of transport in our institutional animal facility for 3 days before the experiments were carried out. All mice were housed in the specific pathogen-free (SPF)-grade individually ventilated cages (IVCs) and maintained under a 12/12 h light/dark cycle at a temperature of 20–26 °C and relative humidity of 40–70%. The mice were provided food and sterilized water ad libitum. All efforts were made to minimize animal suffering. The animal study was approved by the Animal Experimentation Ethics Committee of the Anhui Medical University (approval no. LLSC20230023), and all animals in this study were treated according to the guidelines of Anhui Medical University and with the approval of the Animal Care and Ethics Committee of Animal Usage of Anhui Medical University (Hefei, China).

A modified version of the previously described unobstructed ascending UTI mouse model was employed. To inoculate the mice with bacteria, a bacterial culture was prepared by incubating it overnight at 37 °C in LB broth. The culture was then reinoculated in fresh LB broth and centrifuged at 5000 rpm for 10 min. The resulting pellet was dissolved in 10 ml of sterile PBS. The cell densities were adjusted to 5 × 10^7^ CFUs per 25 μl for the prototypic UPEC strain, CFT073.

After the mice were anesthetized with isoflurane, a syringe covered with a sterile urinary catheter was gently inserted into the urethra. Once the catheter was fully inserted, 50 μl of the bacterial inoculum was slowly injected into the bladder by depressing the syringe plunger. Thirty minutes after urethral inoculation with CFT073, the mice were anesthetized again and, upon regaining their normal mobility, were returned to their original cages.

To ensure the stability of the infection model, the same infection procedure was repeated on the second day. On the third day, the infected mice were subjected to different treatments involving bladder instillation. These treatments included instilling 50 μl of saline, levofloxacin, PADM, and Lev@PADM into the bladder, respectively.

After seven days, the mice were sacrificed. Bladder tissue samples were collected, fixed in 4% (w/v) paraformaldehyde, pre-cooled in liquid nitrogen, and stored at − 80 °C for further processing. Serum and urine samples were also collected and stored at − 80 °C.

### Immunohistochemistry and immunofluorescence double staining of bladder tissue

For histopathology analysis, bladder sections were cut into 4-μm-thick slices and rehydrated using a series of ethanol solutions with increasing concentrations. Antigen retrieval was performed by heating the slides in a microwave at 95 °C for 15 min, followed by cooling to room temperature and washing with Tris-buffered saline containing 0.1% (v/v) Tween^®^ 20 Detergent (TBST) wash buffer. To block non-specific protein binding, the slides were incubated with normal goat serum (Gene Tech). Immunohistochemistry was performed using specific primary antibodies, including anti-iNos (18985-1-AP, Proteintech), anti-CD206 (18704-1-AP, Proteintech), anti-MPO (22225-1-AP, Proteintech), and anti-Mast (18189-1-AP, Proteintech). Immunofluorescent staining was performed using anti-E-cadherin (#BF0219, Affinity Biosciences) and anti-*E.coli* cells (#ab137967; Abcam) antibodies. The slides were incubated with the primary antibodies overnight at 4 °C, followed by washing with TBST. Detection of the target proteins was achieved using either HRP-conjugated secondary antibodies for immunohistochemistry or fluorescent-conjugated secondary antibodies for immunofluorescence staining. Images were captured using a fluorescence microscope (Olympus, Tokyo, Japan) with consistent acquisition parameters.

### Detection of levofloxacin concentration

Mice were treated with 50 μl of levofloxacin solution via oral gavage and bladder injection, respectively, while another group of mice was treated with 50 μl of Lev@PADM hydrogel via bladder instillation. All three intervention groups received an equal dose of levofloxacin (20 μg/ml). Urine samples were collected from the mice at 3/6/24/48 h after treatment, and the concentration of levofloxacin in the urine was determined.

### The impact of Lev@PADM on intestinal microbiota structure

The mice were divided into three groups: control group, levofloxacin oral gavage group, and Lev@PADM bladder instillation group. Each group received the respective intervention treatment continuously for seven days. Fecal samples were collected from the mice on Day 8 and immediately stored at – 80 °C. Total bacterial genomic DNA was extracted from the fecal samples using the QIAamp 96 Powerfecel QIAcube HT Kit (Qiagen, Germany) according to the manufacturer’s instructions. Amplicon sequencing of the V3-V4 region of the bacterial 16S rRNA gene was performed using primers 343F (5’ TACGGRAGGCAGCAG 3’) and 798R (5’ AGGGTATCTAATCCT 3’) on an Illumina HiSeq 2500 System at Personalbio Biotech Co., Ltd. (Shanghai, China). The raw sequencing data were obtained in FASTQ format. Preprocessing of the paired-end reads was performed using Trimmomatic software to detect and remove ambiguous bases (N). Low-quality sequences (average quality score less than 20) were trimmed using a sliding window approach, followed by read assembly using the FLASH software. QIIME software (version 1.8.0) was used for further denoising of the sequences. Operational taxonomic units (OTUs) were defined using the Vsearch software with a 97% similarity cutoff. Representative reads from each OTU were selected using the QIIME package. All representative reads were annotated by blasting against the Silva database Version 138 using the RDP classifier with a confidence threshold of 70%. Analysis of various parameters such as α-diversity and β-diversity of the microbial community was conducted using the GENESCloud platform developed by Personalbio Biotech Co., Ltd.

### Statistical analysis

Data are expressed as mean ± SEM. Statistical significance was evaluated using an unpaired two-tailed Student’s t-test and one-way ANOVA among more than two groups. Differences were considered statistically significant at p < 0.05. GraphPad Prism version 7.0 (GraphPad Software) was used for the statistical analyses.

### Supplementary Information

**Additional file 1: Figure S1.** Self-assembly of decellularized extracellular matrix hydrogel under different pH conditions.

**Additional file 2: Figure S2.** Comparisons of tissue weights after treatment with Lev@PADM and saline. (A) Bright-field images of the kidneys, spleen, and bladder after seven days of treatment with physiological saline and Lev@PADM. (B) Statistical analysis of tissue weights (ns: not significant).

**Additional file 3: Figure S3.** In vivo safety evaluation of Lev@PADM. A TUNEL assay and HE staining were used to assess the integrity of bladder epithelial structures. Scale bar = 200 μm. (B). Quantification of positive cells in the TUNEL assay. (ns: no significant).

**Additional file 4: Figure S4.** Lev@PADM reduces the expression of inflammatory factors by inhibiting the activation of the NF-κB signaling pathway. (A) The protein levels of TLR4, p-P65, p-IκB, TNFα, and IL-1β were detected in the bladder of Saline group, levofloxacin group, PADM group and Lev@PADM group before and after infection. (B) The quantitative data in panel A. The values were expressed as mean ± SEM from 6 mice in each group. *P < 0.05, **P < 0.01, ***P < 0.001.

**Additional file 5: Figure S5.** Immunohistochemical analysis of bladder tissue. (A) The expression of p-P65 was detected by using immunohistochemistry. Scale bar = 200 μm. (B) The quantitative data in panel B. The data were expressed as mean ± SEM from 6 mice in each group. *P < 0.05, **P < 0.01.

## Data Availability

No datasets were generated or analysed during the current study
