# Supplementary figures and images for: Fabrication of levofloxacin-loaded porcine acellular dermal matrix hydrogel and functional assessment in urinary tract infection
Source: J Nanobiotechnology. 2024 Feb 7;22:52. doi: 10.1186/s12951-024-02322-w (PMC10848372; doi:10.1186/s12951-024-02322-w)

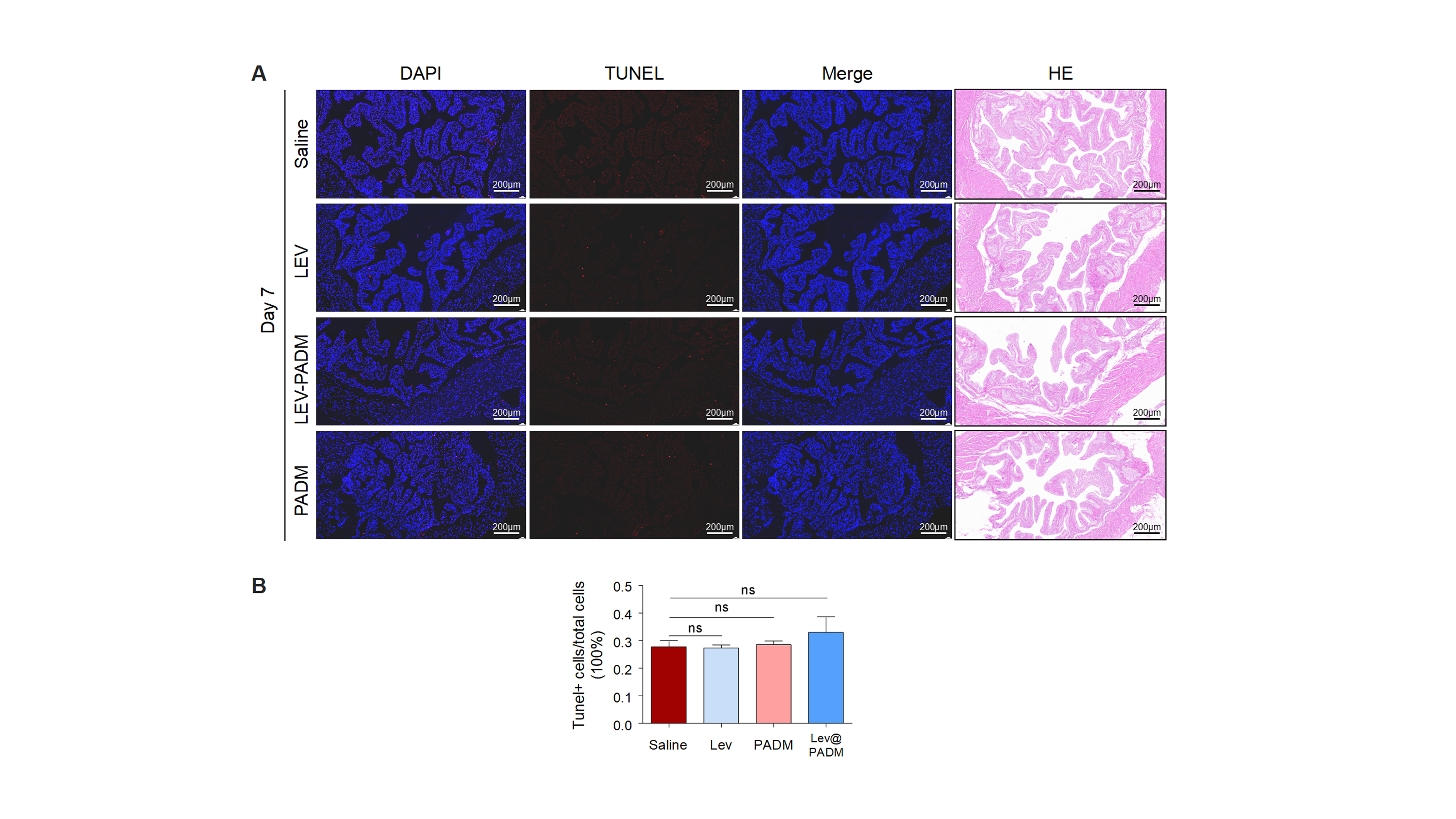

Supplement: Supplementary file 1 — Additional file 1: Figure S1. Self-assembly of decellularized extracellular matrix hydrogel under different pH conditions. [file 12951_2024_2322_MOESM1_ESM.tif]

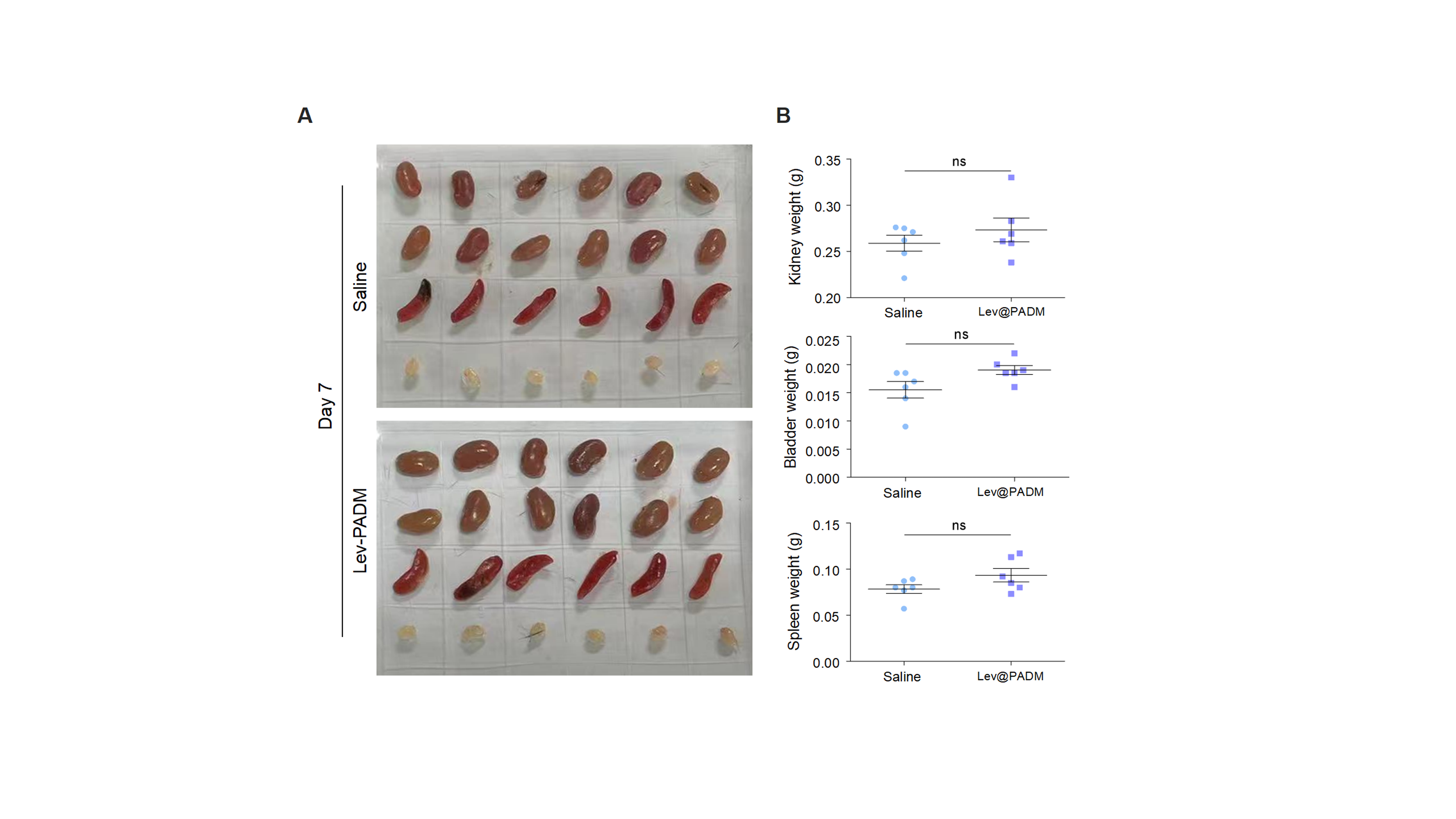

Supplement: Supplementary file 2 — Additional file 2: Figure S2. Comparisons of tissue weights after treatment with Lev@PADM and saline. (A) Bright-field images of the kidneys, spleen, and bladder after seven days of treatment with physiological saline and Lev@PADM. (B) Statistical analysis of tissue weights (ns: not significant). [file 12951_2024_2322_MOESM2_ESM.tif]

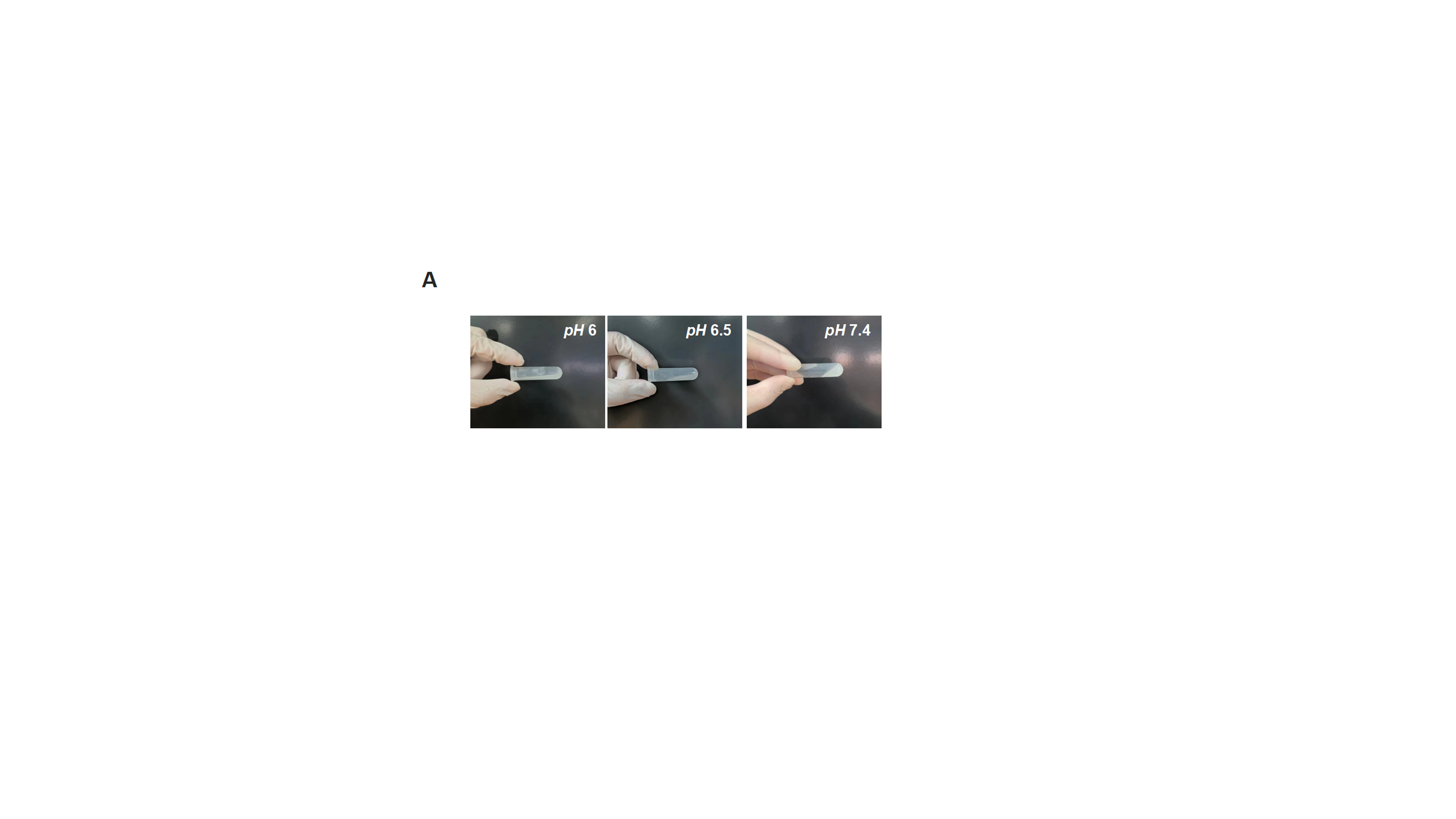

Supplement: Supplementary file 3 — Additional file 3: Figure S3. In vivo safety evaluation of Lev@PADM. A TUNEL assay and HE staining were used to assess the integrity of bladder epithelial structures. Scale bar = 200 μm. (B). Quantification of positive cells in the TUNEL assay. (ns: no significant). [file 12951_2024_2322_MOESM3_ESM.tif]

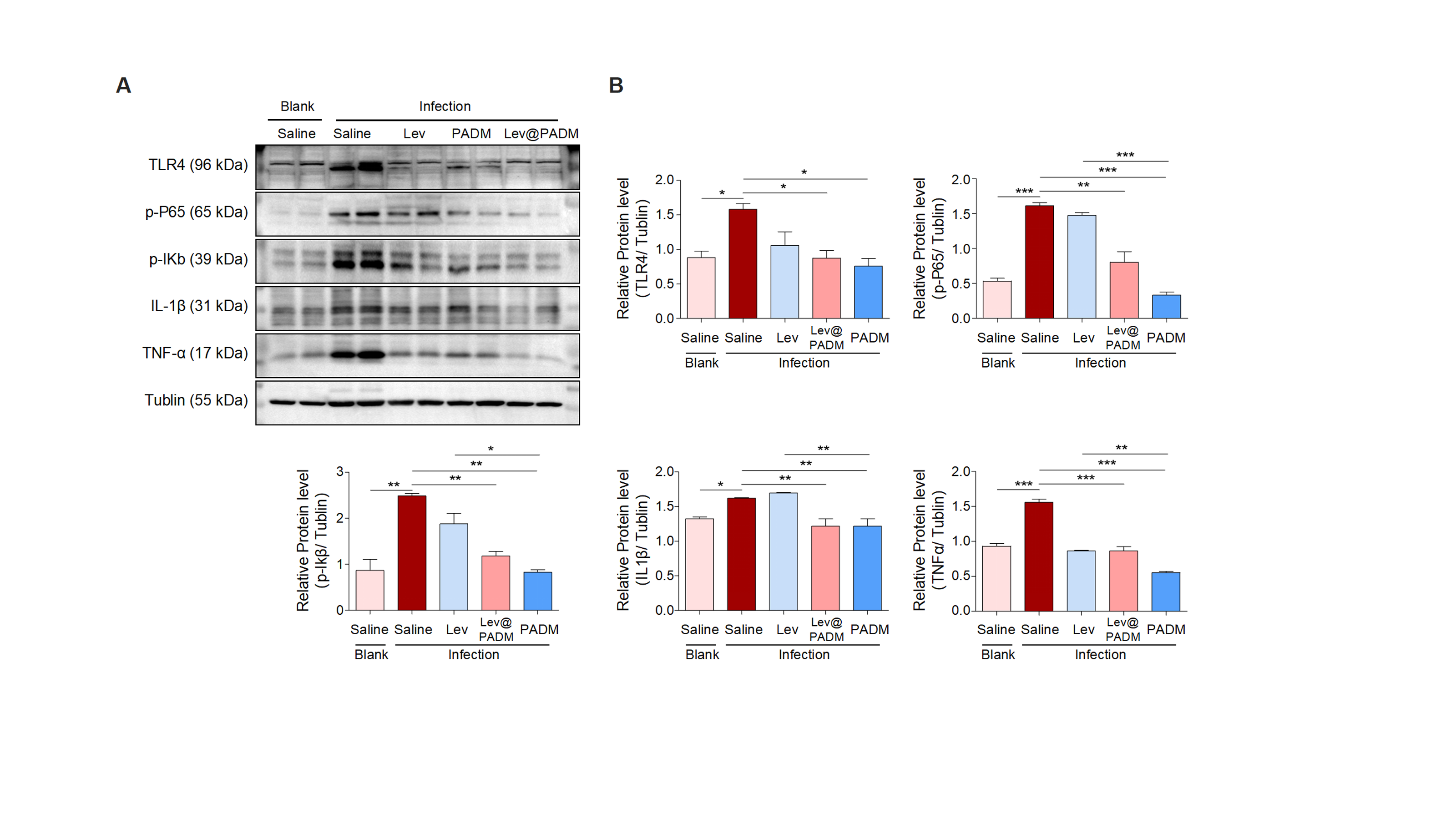

Supplement: Supplementary file 4 — Additional file 4: Figure S4. Lev@PADM reduces the expression of inflammatory factors by inhibiting the activation of the NF-κB signaling pathway. (A) The protein levels of TLR4, p-P65, p-IκB, TNFα, and IL-1β were detected in the bladder of Saline group, levofloxacin group, PADM group and Lev@PADM group before and after infection. (B) The quantitative data in panel A. The values were expressed as mean ± SEM from 6 mice in each group. *P < 0.05, **P < 0.01, ***P < 0.001. [file 12951_2024_2322_MOESM4_ESM.tif]

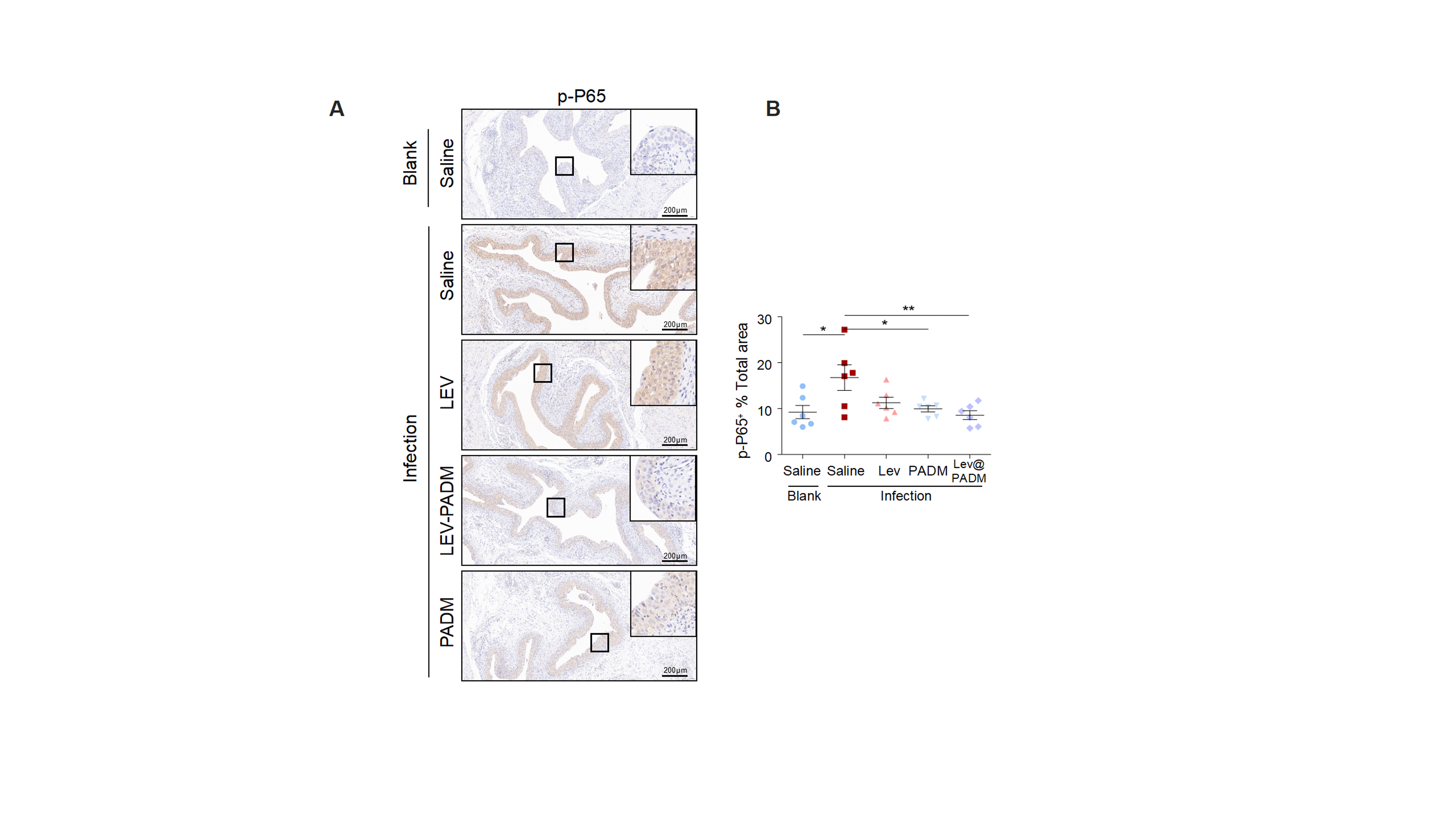

Supplement: Supplementary file 5 — Additional file 5: Figure S5. Immunohistochemical analysis of bladder tissue. (A) The expression of p-P65 was detected by using immunohistochemistry. Scale bar = 200 μm. (B) The quantitative data in panel B. The data were expressed as mean ± SEM from 6 mice in each group. *P < 0.05, **P < 0.01. [file 12951_2024_2322_MOESM5_ESM.tif]
